# The Influence of Epidemiologic Context on the Success of Partner Notification Programs: Analysis of Gonorrhea Transmission Dynamics

**DOI:** 10.1093/infdis/jiaf206

**Published:** 2025-04-18

**Authors:** Minttu M Rönn, Harrell W Chesson, Yonatan H Grad, Marissa Reitsma, Lin Zhu, Katherine Hsu, Thomas L Gift, Joshua A Salomon

**Affiliations:** Department of Global Health and Population, Harvard T.H. Chan School of Public Health, Boston, Massachusetts; Division of STD Prevention, Centers for Disease Control and Prevention, Atlanta, Georgia; Department of Immunology and Infectious Diseases, Harvard T.H. Chan School of Public Health, Boston, Massachusetts; Department of Health Policy, Stanford University, California; Department of Health Policy, Stanford University, California; Sexually Transmitted Disease Prevention and HIV/AIDS Surveillance, Massachusetts Department of Public Health; Section of Pediatric Infectious Disease, Boston Medical Center, Massachusetts; Division of STD Prevention, Centers for Disease Control and Prevention, Atlanta, Georgia; Department of Health Policy, Stanford University, California

**Keywords:** gonorrhea, men who have sex with men, network model, partner notification, sexually transmitted infections

## Abstract

**Background:**

Limited estimates exist on the population-level impact of partner notification (PN) for gonorrhea, with uncertainty in the influence of local epidemiology on PN effectiveness. An ecologic study in New York found a 6% reduction in diagnoses with a 10% increase in PN coverage. We estimated gonorrhea incidence reductions via PN across different epidemiologic conditions to compare the effects with the prior finding and to understand key determinants of variation.

**Methods:**

We developed a stochastic network model of men who have sex with men and calibrated gonorrhea transmission dynamics to varied epidemiologic conditions. The population-level impact of increasing PN was summarized by incidence rate ratios (IRRs), and the relative importance of explanatory variables (including network density, baseline burden, and natural history parameters) was assessed via linear regression modeling of IRR and bootstrapping to evaluate uncertainty in estimation.

**Results:**

We estimated an IRR of 0.97 (95% range, 0.93–0.99) for a 10% relative increase in PN coverage, comparable to the IRR of 0.94 (0.91–0.97) identified in the empirical study. PN retained effectiveness under diverse epidemiologic conditions. In a univariate sensitivity analysis, the strongest influence on IRR came from parameters governing index case testing probability, with an IRR of 0.93 when testing was at its highest. Other factors, such as network density, baseline incidence, and various natural history parameters, had relatively minor effects on the IRR. We observed larger individual-level benefits from PN for individuals with higher numbers of partners.

**Conclusions:**

Our findings support prior population-level estimates of the impact of PN on gonorrhea incidence.

## Teaser

We estimated an incidence rate ratio of 0.97 (95% range, 0.93–0.99) for a 10% relative increase in partner notification coverage. Our findings support prior population-level estimates of the impact of partner notification on gonorrhea incidence.

Gonorrhea prevention in the United States has traditionally focused on screening persons at risk for gonorrhea, treating those who are infected, and treating their sex partners who are potentially infected [1]. Partner notification promotes the treatment of partners who are infected by identifying and informing the sex partners of individuals with confirmed infections and facilitating their access to testing and treatment.

Clinical trials have demonstrated the effectiveness of partner notification strategies in reducing an index patient's risk of reinfection with gonorrhea and other curable sexually transmitted infections; however, the potential effect of partner notification on gonorrhea incidence at the population level is more difficult to measure [2, 3]. Clinical trials are poorly suited for estimating the population-level impact of partner notification. The trials would need to be sufficiently large to influence the transmission dynamics of the broader sexual network, and they would have to focus on partner notification implementation strategies, with both trial arms offered some form of partner notification (eg, expedited partner treatment vs traditional partner referral) [2]. In trials, intermediate process-level measures are often used as outcome measures to estimate effects of the trial [3]. The most common measure of transmission dynamics is reinfection rate in the index cases. For example, expedited partner treatment (vs traditional partner notification) was estimated to reduce reinfection in index cases for curable sexually transmitted infection with a pooled relative risk of 0.71 (95% CI, 0.56–0.89) [2]. Although reinfection risk gives an indication of sexually transmitted infection transmission risk among close partners of the trial participants, it does not quantify the population-level impact of partner notification [2, 3].

Estimates of the population-level impact of partner notification on gonorrhea incidence have instead been derived through ecologic study. An analysis of surveillance data in New York State found an association between increasing partner notification and decreasing gonorrhea diagnosis rates at the county level [4]. Du et al estimated that gonorrhea diagnoses were reduced by 6% for every 10% increase in partner service coverage. At this effect size, increases in partner notification coverage have been estimated to be cost saving [5]. However, as an ecologic study, the findings are subject to limitations. For example, the study was based on surveillance data of gonorrhea diagnoses from 1992 to 2002, a period when gonorrhea diagnoses in the United States were in decline in part due to behavioral changes attributed to HIV [6]. Thus, it is possible that the impact of partner notification was overestimated in the observational data examined by Du et al.

Our study aims to contribute to the evidence base of the effectiveness of partner notification. First, we evaluated the reproducibility of findings from the Du et al study [4] by modeling whether an increase of 10% in partner notification efforts could plausibly reduce gonorrhea incidence by 6%. Second, we examined the relative importance of network characteristics and epidemiologic context on the impact of partner notification.

The analyses used a network model of gonorrhea transmission among men who have sex with men (MSM), a community disproportionately affected by gonorrhea in the United States. The model structure was kept parsimonious, and it was calibrated over a range of epidemiologically plausible ranges by varying the key input parameters to capture a wide set of epidemiologic conditions and parameter combinations. The study has the potential to improve gonorrhea control programs by identifying contextual factors that can influence the effectiveness of partner notification, using gonorrhea transmission among MSM as an example.

## METHODS

### Mathematical Model

We simulated sexual networks and gonorrhea transmission dynamics and natural history among a population of 2000 MSM (population size defined by balancing model run time and stability of findings). Sexual ties (sexual partnerships) included main, casual, and one-off partnerships. *Main partnership* represents regular partners, which describes established partnerships with more frequent sexual contact, while *casual partnership* represents known partners with occasional sexual contact. Main and casual partnerships are modeled with static ties—specifically, these partnerships were present for the duration of the analysis, as opposed to dynamic ties, which are defined by formation and dissolution of a partnership. We modeled one-off partnerships as a subset of individuals having the propensity for one-off partnerships. These persons may form one-off partnerships with each other, and these are modeled as instantaneous partnership formation and dissolution. Networks were generated by exponential random graph models [7]. We generated sexual networks with lower and higher numbers of partnerships between individuals (lower- and higher-degree networks). Details are in the supplementary material.

The natural history of gonorrhea was modeled as a stochastic susceptible-infected-susceptible process. For partnerships represented by static ties (casual and main partners), we modeled transmission as a combined probability, considering the probability of contact (frequency of sexual activity) and transmission if the partner is infected. Main partnerships were assigned a higher weekly probability than casual partnerships based on more frequent contact (Table 1). We modeled transmission for one-off partnerships differently, whereby we used a per-week probability of a person forming a one-off partnership. Given that a one-off partnership occurs, transmission probability per partnership was modeled as a Bernoulli process, accounting for the number of acts per partnership, the transmission probability per act, and the prevalence of infection among individuals engaging in one-off partnerships. Gonorrhea clearance could occur via natural clearance or through treatment following testing due to symptoms or screening of persons with asymptomatic infections, each with weekly probabilities. We modeled infection at the individual level and did not consider site of infection, given the challenges associated with accurately modeling transmission dynamics across multiple sites of infection [14].

**Table 1.** Parameters Used in the Transmission Model: Base Case Values and 95% Range, Prior Distribution, Parameter Description, and Data sources

	Prior Distribution			
Parameter Name	Parameter (95% Range)	Informed by Data	Variable	Source
Population size	2000 MSM	Fixed	No. of MSM in the model	Authors’ discretion
Time step	1 wk	Fixed	Time step used in analysis	Authors’ discretion
Time horizon	8 y	Fixed	4-y burn-in period, 4-y evaluation	Authors’ discretion
One-off partnership				
Proportion of people who engage in one-off partnerships in the population	0.47 (0.40–0.54)	Beta (90, 100)	Proportion of people who engage in one-off partnerships, distributed randomly across the model individuals (independent of the number of main and casual partners).	[8, 9]
Weekly probability of engaging in one-off partnership	0.16 (0.15–0.18)	Beta (593.10, 3045.57)	The mean rate of onetime partnership acquisition was 0.163/wk (95% CI, 0.152–0.175), which is ∼8.5 partners/y	[8]
No. of acts per one-off partnership	2 (1–5)	1 + Poisson (1.5)	No. of sexual acts per one-off partnership. Assumption: minimum of 1 act.	Assumption
One-off partners: probability per act	0.01 (0.00-0.03)	Beta (2, 200)	Probability of transmission per act in one-off partnership. No. of acts is defined separately	Assumption
Probability of contact and transmission probability: combined parameter for static partnerships^a^				
Casual partners: weekly probability	0.01 (0.00-0.03)	Beta (2, 200)	Probability of contact between casual partners * probability of transmission if partner infected	Assumption
RR for main partners	1.67 (1.01–2.93)	1 + gamma (2,2.9)	RR to casual partners to account for more frequent contact between main partners	Assumption
Main partners: weekly probability	0.0409 (0.04-0.212)	Derived	Casual partner parameter * RR for main partners.	Derived
Natural history				
Probability of symptomatic infection	0.41 (0.17–0.69)	Beta (5.05, 7.05)	Probability that an infection is symptomatic. Reflects an average (across sites of infection)	[10, 11]
Natural clearance probability	0.07 (0.04–0.18)	Beta (14.12, 172.70)	Weekly probability of spontaneous clearance. Used the duration of rectal gonorrhea as guidance in setting up clearance probability	[12]
Testing and treatment				
Symptomatic infection	0.0812 (0.005–0.325)	Beta (1.19, 10.28)	Weekly probability of testing in persons who develop symptoms. Generic male symptomatic duration used to inform prior distribution	[10]
Asymptomatic infection	0.019 (0.002–0.074)	Beta (1.54, 60.91)	Weekly probability of testing in persons without symptoms. Lower level from Kreisel et al (16%/y, middle point to 1/y, and upper limit 4 times/y)	[10, 13]
Partner notification				
Index interview	0.42 (0.34–0.538)	Beta (39.84, 51.69)	Probability that an index case is interviewed. Du et al: 0.30–0.55 taken as IQR	[4]
Partner reached	0.85 (0.77–0.91)	Beta (80.10, 14.84)	Probability that a partner is reached if partner notification is initiated. Du et al: 0.8–0.9 taken as IQR	[4]
Partner notification to main partner	Derived 0.37 (0.28-0.46)	Derived	Index interview * partner reached. Assumed only main partners receive partner notification at baseline	Derived

Abbreviations: MSM, men who have sex with men; RR, risk ratio.

^a^The parameter includes the probability of contact and the probability of transmission if the partner is infected.

We applied conservative assumptions that were biased against partner notification: (1) index cases (persons diagnosed and selected for partner notification) could notify 1 main partner if they have at least 1 main partner, and (2) index cases could not notify casual partners or one-off partners. We included partner notification in the baseline model and modeled partner notification coverage as a function of index cases whose partners receive notification, defined as the ratio of the number of partners notified to the number of index cases diagnosed. To reduce stochastic variability and to allow a more direct simulation-level comparison, common random numbers [15] were implemented for all events that occurred at the individual level or in partnerships. A description is in the supplementary material.

### Calibration

Network type (lower- or higher-degree network), parameters related to partnerships, natural history parameters, testing of index cases, and partner notification coverage were varied in calibration. In total, 12 parameters were varied in calibration (Table 1). We calibrated the model using target fitting and broad ranges for calibration to cover a range of epidemiologic contexts, including lower and higher burden, and varied testing frequency for gonorrhea, which can result in different diagnosis rates and the proportion identified with symptoms (Supplementary Table 1). Calibration ranges were prevalence (target, 2%–10%), annual rate of incident infections per population (2%–23%), annual rate of diagnoses per population (1%–10%), and proportion of index cases that were symptomatic when diagnosed (30%–80%).

Given the broad and multidimensional parameter space, target fitting was implemented in 2 steps. In the first stage, we sampled parameter values from their prior distributions and simulated the model 10 times per parameter set. Allowing for “burn-in” period, we calculated mean outcomes across the 10 simulations after 4 years and retained “candidate” parameter sets with mean outcomes falling within target ranges. In the second stage, we ran 80 simulations for each candidate parameter set based on an analysis of the number of simulations needed to achieve stable estimates with common random numbers (Supplementary Figure 3). In the second stage, we retained parameter sets from the subset of candidates based on the mean outcomes across 80 simulations falling within the target ranges.

### Analysis

Our first aim was to test the replicability of the effect size estimate from the empirical study in New York by Du et al [4]. The Du et al study reported the reduction in the gonorrhea diagnosis rate associated with a 10% increase in partner services but did not explicitly clarify whether the increase was a 10% relative or 10% absolute increase. To compare our results with the Du et al study's effect size, we examined a 10% relative increase and a 10% absolute increase in partner services as compared with baseline. The baseline level of partner notification was determined by specifying a prior distribution on coverage with a mean 0.37% (95% range, 0.28%–0.46%; as informed by Du et al). We also examined a 20% and 40% increase in partner notification. We calculated the incidence rate ratio (IRR) as the primary outcome measure, as analogous to the Du et al diagnosis rate ratio. IRR is the ratio of incidence in the increased partner notification simulation to the incidence in the paired baseline simulation in the same year. IRR was calculated at the simulation level and averaged at the parameter set level (across 80 simulations from 1 parameter set).

Our second aim was to evaluate key drivers of variation in effect size estimates of partner notification. We constructed a linear regression model with the IRR for a relative 10% increase in partner notification as the dependent variable, as well as an array of independent variables, including 4 network-level variables and 10 additional model parameters used for calibration. Network-level parameters included lower or higher degree of network (categorical variable, higher degree as the reference), baseline incidence, proportion of ties that are main partnerships, and proportion of individuals who engage in one-off partnerships. Other parameters that were varied during model calibration reflected uncertainty in the natural history and intensity of prevention services at baseline: probability of symptomatic infection if infected, weekly probability of natural clearance, weekly probability of testing if symptomatic infection, weekly probability of testing if asymptomatic infection, baseline coverage of partner notification, probability of contact and transmission for main and casual partners, weekly probability of engaging in one-off partnership, transmission probability per one-off act, and number of acts per one-off partnership. The analysis was done at the parameter set level (eg, baseline incidence was computed as the average across simulations in each parameter set). We used bootstrapping to account for uncertainty in the stability of the estimates given variation in calibrated parameter sets included in the study, generating 50 000 samples of the inputs and outputs for the regression model with replacement from the full analytic data set. We used the regression model to construct a modified tornado plot examining how the IRR changes as each independent variable is assigned its maximum or minimum value while all other variables are held at their mean values.

To examine the broader effects of partner notification within the population, we calculated averted infections over the 4-year follow-up after partner notification was increased. Averted infections were calculated at the individual level per simulation, aggregated by the individual’s main and casual partner number, and averaged across simulations. The model was coded in R. Analytic code is at https://github.com/mintturonn/gonorrhea-pn-determinants.

## RESULTS

We identified 415 candidate parameter sets in the first step of calibration and retained 243 calibrated parameter sets at the second step. These included 172 parameter sets with the higher-degree network and 71 parameter sets with the lower-degree network. Calibration figures are presented in Supplementary Figure 4, parameter prior and posterior distributions are in Supplementary Figure 5, and correlation between the parameters included in the regression model is in Supplementary Figure 6.

**Figure 1.**
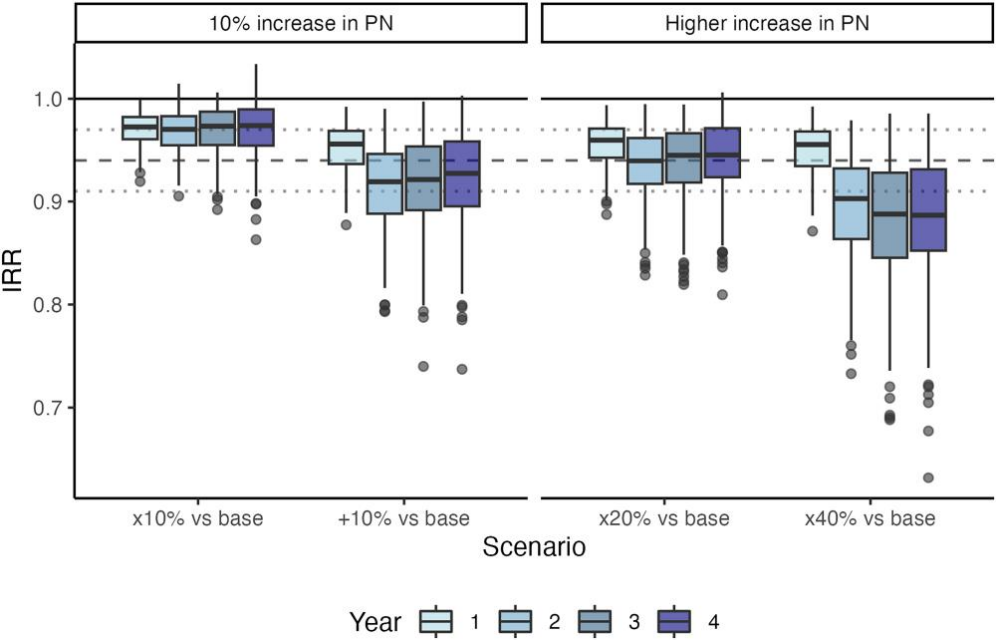
Incidence rate ratio (IRR) by year of follow-up, shown as a Tukey box plot. Dashed horizontal line represents the rate ratio reported in the Du et al study [4] and dotted lines the 95% CI. The 10% increase, relative (×10%) and absolute (+10%), is presented with higher relative increases in partner notification (PN), with ×20% for the 20% relative increase to baseline PN (base) and ×40% for 40%. The IRR shows the ratio of gonorrhea incidence in a scenario of increased PN as compared with baseline levels of PN. A lower IRR reflects a greater impact of PN. The Tukey box plot shows the median (IQR) with “whiskers” indicating a range from smallest to largest values within 1.5 times the IQR and with outliers presented as dots.

Our results aligned with the confidence intervals of the Du et al study [4] (Figure 1), which reported a diagnosis rate ratio of 0.94 (95% uncertainty interval [UI], 0.91–0.97) with a 10% increase in partner notification. An IRR of 0.94 indicates a 6% reduction in incidence. In a population of 1000 with a baseline incidence of 10%, this means 6 fewer infections, decreasing from 100 to 94 infections. Variation in the modeled IRR estimates reflects parametric uncertainty (IRRs are computed from the average of the simulations for each calibrated parameter set). Across the parameter space examined, we observed varying effect sizes with a 10% relative increase in partner notification increase, with a median IRR of 0.97 (95% range, 0.93–0.99) in the first year. There is a more pronounced impact with a 10% absolute increase, although the estimates fall within the confidence intervals of the Du et al study. The benefits grow as partner notification coverage expands. For instance, with a 20% increase in partner notification relative to the baseline, the IRR (95% UI) in the first year was 0.96 (0.91–0.99) and 0.94 (0.85–0.98) in the second year; a 40% increase relative to the baseline resulted in a first-year IRR of 0.96 (0.89–0.98) and a second-year IRR of 0.90 (0.78–0.97).

**Figure 2.**
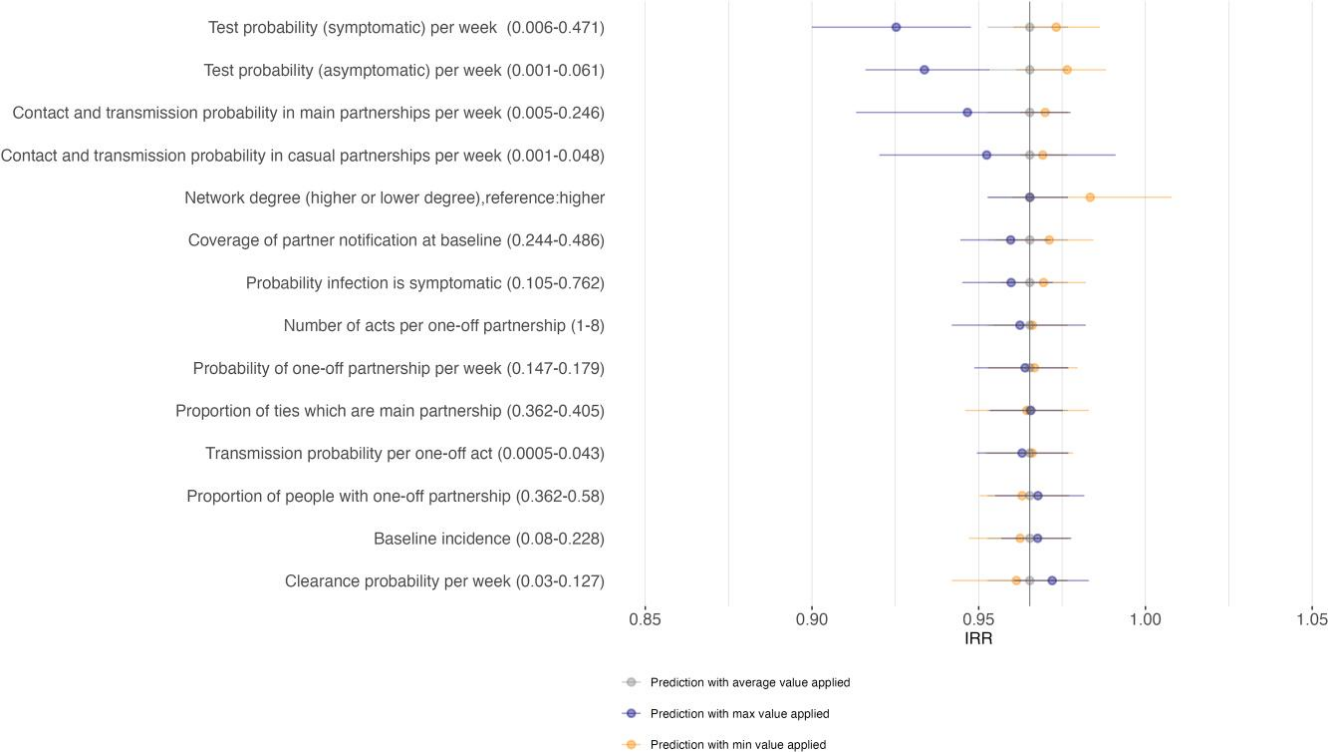
Tornado plot illustrating the predicted incidence rate ratio (IRR) and associated uncertainty following a 10% relative increase in partner notification. The plot displays results from bootstrapping samples from the full data and repeating regression analyses with subsets of the data. The vertical line represents the predicted IRR based on the average of the data, and the minimum and maximum prediction is done by using the calibrated model values (full range shown in brackets). The uncertainty is shown as the average prediction (point) and the 100% range in the prediction (vertical lines). The IRR shows the ratio of gonorrhea incidence in a scenario of increased PN as compared with baseline levels of PN. A lower IRR reflects a greater impact of PN.

The uncertainty in the estimates of the IRR increases over time as the simulations accumulate more variability in events and trajectories, diminishing the reliability of determining the driving factors. To identify key influences, we focused on explanatory variables on the first-year IRR following a 10% relative increase in partner notification. The regression model predicts an IRR 0.97, which corresponds to the simulation model results with an *R*^2^ value of 63.7%. The modified tornado plot in Figure 2 illustrates the relative importance of each explanatory variable in the regression model when accounting for uncertainty in these estimates using bootstrapped samples. The strongest influence in variation of IRR came from parameters governing testing probability (weekly testing probability due to symptomatic or asymptomatic infection), where an increased probability of index case testing correlated with a larger decrease in IRR. Based on the maximum values for testing symptomatic and asymptomatic cases, the IRR (95% UI) was 0.93 (0.90–0.95) and 0.93 (0.92–0.95), respectively. According to their minimum values, the IRR estimates were 0.97 (0.96–0.99) and 0.98 (0.96–0.99), and the minimum values overlapped with the average prediction. For other variables examined, the uncertainty intervals overlapped with the average prediction. Contact frequency and transmission probability parameters for main and casual partners were associated with the most uncertainty. Given the influence of index case testing on IRR, we used the regression model to estimate predicted IRR in the absence of screening, and IRR was predicted at 0.98 with a 10% relative increase in partner notification. When we additionally assumed that the probability of testing due to symptoms was at the maximum of the calibrated model estimates (92% of people with symptoms get tested within a month of acquiring infection) and there was no screening in the population, the predicted IRR was estimated at 0.93.

**Figure 3.**
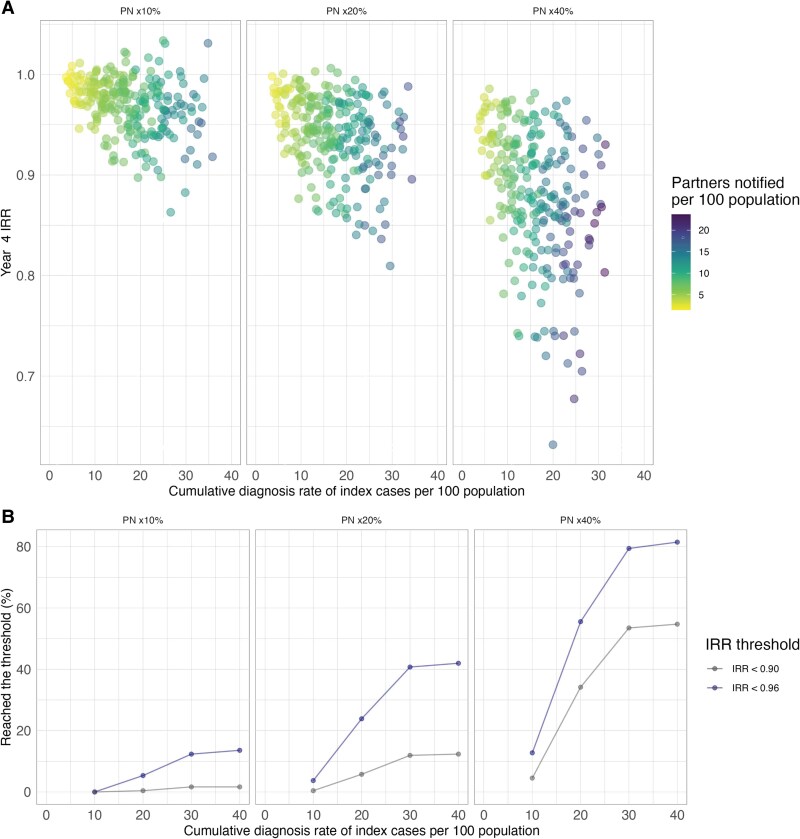
Incidence rate ratio (IRR) on the fourth year of intervention when compared against the cumulative gonorrhea diagnosis rate for index cases (x-axis) and cumulative rate of partners notified and diagnosed (represented with color). *A*, Each dot presents the average of a calibrated parameter set. *B*, The cumulative percentage (y-axis) of parameter sets predicts an IRR below 2 illustrative threshold values (IRR <0.90 and IRR <0.96) given the index case diagnosis rate during the intervention period. The IRR shows the ratio of gonorrhea incidence in a scenario of increased PN as compared with baseline levels of PN. A lower IRR reflects a greater impact of PN. The index cases diagnosed (combining index cases identified due to symptom-based testing and screening) and the partners notified and diagnosed present the 4-year cumulative rate per 100 total population in the intervention scenario based on the total population modeled as the denominator.

The relationship among index case finding, number of partners notified, and resulting changes in predicted IRR is illustrated in Figure 3. Overall, 14% of calibrated parameter sets predicted an IRR <0.96 in the fourth year of the intervention when partner notification increased 10% relative to baseline, 42% when partner notification increased 20% relative to baseline, and 82% when partner notification increased 40% relative to baseline. The predicted incidence reduction with a 40% increase in partner notification at the lowest levels of the index case diagnosis rate was comparable to the incidence reduction with a 10% increase in partner notification at the highest levels of the index case diagnosis rate: 13% and 14% of parameter sets predicted an IRR <0.96, respectively. The combined impact of index case finding and partner notification in reducing incidence can be illustrated by examining the impact of increasing partner notification when examining the same levels of the index case diagnosis rate achieved: when the 4-year cumulative diagnosis rate is up to 20 per 100 population, 5.8% of parameter sets predicted an IRR <0.90 in the 20% increase in the partner notification scenario, while in the 40% increase scenario, 34.2% of parameter sets predicted an IRR <0.90.

**Figure 4.**
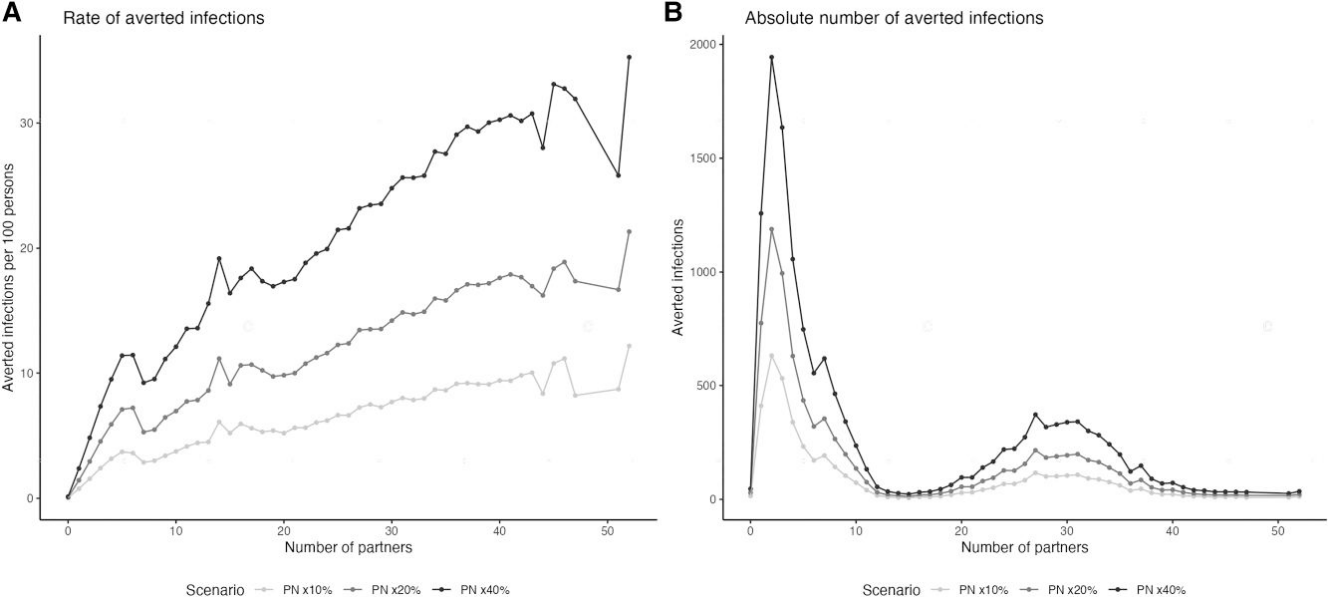
Mean cumulative number of averted infections (mean across simulations) over a 4-year time frame at the individual level stratified by number of casual and main partners that individuals had. *A*, Rate of cumulative infections per population by partner number. *B*, The absolute number of infections averted by partner number. Three scenarios are shown to illustrate different relative increases (10%, 20%, and 40%) in partner notification (PN) coverage relative to baseline levels.

We observed larger individual-level benefits from partner notification as individuals’ numbers of main and casual partners increased. Individuals with the most partners experienced the highest rate of averted infections per population size (Figure 4*A*). However, the largest number of infections was averted among individuals with the lowest number of partners (Figure 4*B*), reflecting this population representing the largest share of the network population size overall (Supplementary Figure 7).

## DISCUSSION

Partner notification could be effective under a range of epidemiologic conditions, pointing to its potential as a versatile intervention strategy for gonorrhea. Our model-based findings on the effect size achievable from partner notification were consistent with findings from the ecologic analysis by Du et al [4], which has been influential in estimating the population-level impact of partner notification in the United States [5]. We estimated that the effectiveness of partner notification in reducing gonorrhea incidence was most influenced by the ability to identify index cases. Symptom-based testing and screening were influential parameters, while other parameters explored did not have such a substantive impact. This superficially obvious finding showcases the robustness of the intervention in the face of extraneous factors, such as the type of sexual network in which gonorrhea is being transmitted or the baseline burden of gonorrhea. Even in the absence of screening, when symptomatic testing was the only mode of identifying index cases, the regression model estimated an IRR of 0.98 in the presence of a modest 10% relative increase for partner notification.

While partner notification can present a robust and effective intervention, the challenges come from implementation. Resource constraints, lack of trust in public health professionals, and stigma are some of the barriers to improved partner notification [16]. We assumed that if partner notification was implemented, a partner with infection was treated during the same week as the index case. Traditional modes of partner notification may achieve this. Recent advances in digital partner notification tools demonstrate scope for further improvements [17]. Expedited partner therapy could also improve population-level outcomes among MSM [18].

There is a growing debate around whether to screen for asymptomatic infections in MSM [19]. Partner notification also identifies asymptomatic infections. This analysis focused on reduction in gonorrhea incidence as the main outcome of interest, assuming that this is a main goal of gonorrhea prevention. We developed a parsimonious model that could be calibrated across a range of epidemiologic contexts and could account for parametric uncertainty. We did not model site-specific infection or testing practices, due to a lack of specific data and the need for a simple model. Nonurogenital infection contributes to gonorrhea burden [20], and more research is needed in this area. The flexible nature of our modeling framework facilitated its calibration across a spectrum of epidemiologic scenarios and multiple parameter combinations. By incorporating multiple parameter combinations, our model encapsulates a greater degree of uncertainty as compared with most conventional network models. Static networks may underestimate real-world transmission dynamics [21] and the true impact of partner notification. The study might underestimate the impact of partner notification by limiting notification to main partners only, and it might overestimate the impact of partner notification by assuming full compliance when implemented. To address concerns over the difficulty of providing partner notification for anonymous contacts, we analyzed partner notification for main partners only, resulting in a conservative effect size estimate of partner notification. Despite partner notification offered to main partners only, we found that increasing coverage in partner notification disproportionately benefits individuals with the highest number of partners, who also have the highest number of casual partners. Improvements in the implementation of partner notification, such as extending notification to multiple partners, including casual partners, would likely yield a greater impact. Our study underlines that partner notification's success relies on index case finding. While testing practices may differ, especially in heterosexual populations, we believe that our key findings remain robust.

While partner notification presents an effective prevention tool, the challenges come from implementation on the optimal strategies to identify index cases and from implementation of partner notification once the index case has been identified. Our study framework offers an improved understanding about the contextual factors that influence the effectiveness of partner notification, and the results can be used for benchmarking the achievable impact across a range of settings. Further research can focus on facilitators that can improve partner notification.

## Supplementary Material

jiaf206_Supplementary_Data

## Supplementary Data

Supplementary materials are available at *The Journal of Infectious Diseases* online (http://jid.oxfordjournals.org/). Supplementary materials consist of data provided by the author that are published to benefit the reader. The posted materials are not copyedited. The contents of all supplementary data are the sole responsibility of the authors. Questions or messages regarding errors should be addressed to the author.
