# Supplementary material for: The Influence of Epidemiologic Context on the Success of Partner Notification Programs: Analysis of Gonorrhea Transmission Dynamics
Source: J Infect Dis. 2025 Apr 18;232(2):e266–74. doi: 10.1093/infdis/jiaf206 (PMC12349959; doi:10.1093/infdis/jiaf206)
Supplement: jiaf206_Supplementary_Data [file jiaf206_supplementary_data.docx]

Supplemental material for

**The influence of epidemiological context on the success of partner notification programs: analysis of gonorrhea transmission dynamics**

Minttu M Rönn^1^, Harrell W Chesson^2^, Yonatan H. Grad^3^, Marissa Reitsma^4^, Lin Zhu^4^, Katherine Hsu^5,6^, Thomas L Gift^2^, Joshua A Salomon^4^

1. Department of Global Health and Population, Harvard T.H. Chan School of Public Health, Boston, MA
2. Division of STD Prevention, Centers for Disease Control and Prevention, Atlanta, GA
3. Department of Immunology and Infectious Diseases, Harvard T.H. Chan School of Public Health, Boston, MA
4. Department of Health Policy, Stanford University, Stanford, CA
5. Sexually Transmitted Disease Prevention & HIV/AIDS Surveillance, Massachusetts Department of Public Health, Boston, MA
6. Section of Pediatric Infectious Disease, Boston Medical Center, Boston, MA

**Corresponding author:** Minttu Rönn, [mronn@hsph.havard.edu](mailto:mronn@hsph.havard.edu)

**Funding:** This work was supported by the U.S. Centers for Disease Control and Prevention, National Center for HIV/AIDS, Viral Hepatitis, STD, and TB Prevention Epidemiologic and Economic Modeling Agreement (5NU38PS004651)

**Disclaimer:** The findings and conclusions in this report are those of the authors and do not necessarily reflect the official position of the Centers for Disease Control and Prevention, or the authors’ affiliated institutions.

**METHODS**

**Static network model**

We modeled three types of partnerships in the population:

1. Main, represented by static network ties, partner notification is only provided between main partners.
2. Casual, represented by static network ties.
3. One-off partnerships, which are not represented by static ties. An instantaneous partnership can be formed by people with a nodal attribute for one-off partnerships.

The modeled network population size is 2,000, and the target statistics are defined based on the division of partnerships (e.g. mean degree) between main, casual and one-off partnerships. Based on data the number of edges were divided between main and casual partners, and we used the total partner number as a nodal attribute to achieve the overall trend in partnership at the population level. We had defined propensity for one-off partnerships as a vertex attribute. These partnerships are not presented by a tie, but they are modeled as instantaneous partnerships, which are drawn randomly from the population of individuals with one-off partnerships.

The following model specifications were used in R.

To form low degree networks the following model specification was used

reg_nw <- ergm(nw ~ edges, target.stats = 450)

cas_nw <- ergm(nw ~ edges, target.stats = 740)

This was informed by a study examining ongoing partnerships,^1^ and represents a more conservative estimate of the number of active ties in the network. In the study, the main partner mean degree was 0.45 (0.43-0.47), and casual mean degree was 0.74 (0.72-0.77).

To form higher degree networks, the population was further stratified by risk

reg_nw <- ergm(nw ~ edges+ nodefactor("activity "), target.stats = c(2490, 697, 588, 1078))

cas_nw <- ergm(nw ~ edges+ nodefactor("activity "), target.stats = c(3320, 1627, 588, 660))

Where risk group was added to generate a more skewed distribution of partnerships (with few individuals having most partners). We included four activity categories (proportion of the population assigned in different activity populations): highest activity (10%), high activity (20%), medium activity (20%) and lowest activity (50%). In the ergm model, the highest risk was set as the reference category. The numbers of partners were informed by number of partners reported among men who have sex with men (MSM) in the past 12 months in the General Social Survey,^2^ and we reduced the number of partners represented by casual and main ties to account for one-off partnerships which were additive.

We generated 10 networks from both lower and higher degree networks. To construct the full sexual network, casual and main networks were combined; if both ties (casual and main) were present between two individuals, the tie was assigned to be casual.

Reporting of one-off partnerships was fairly evenly distributed by partner number,^1,2^ and we implemented this as a nodal attribute that was randomly allocated to the population.


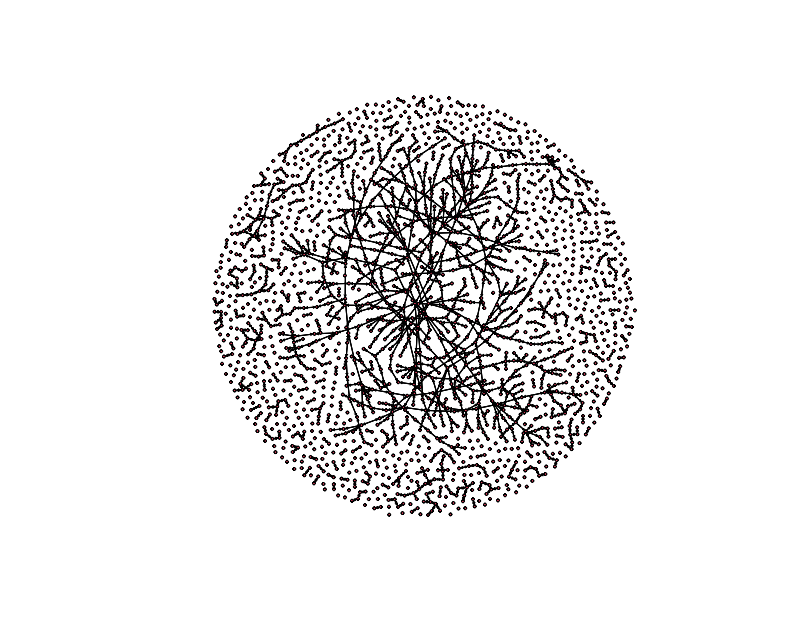
**Figure S1.** Example of lower degree network.

A) Network structure B) distribution of main partners in static network C) number of partners by quantile

**
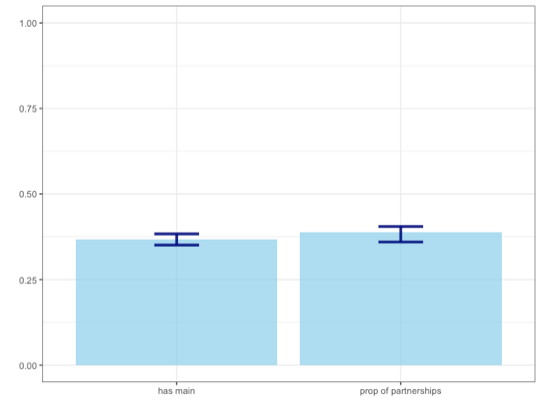
A B**


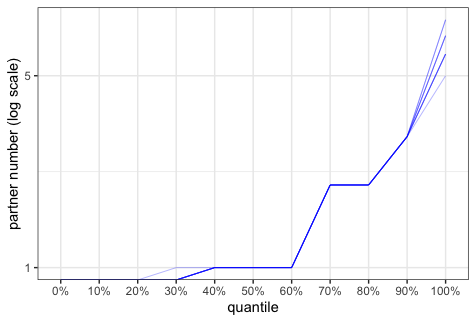
 **C**

**
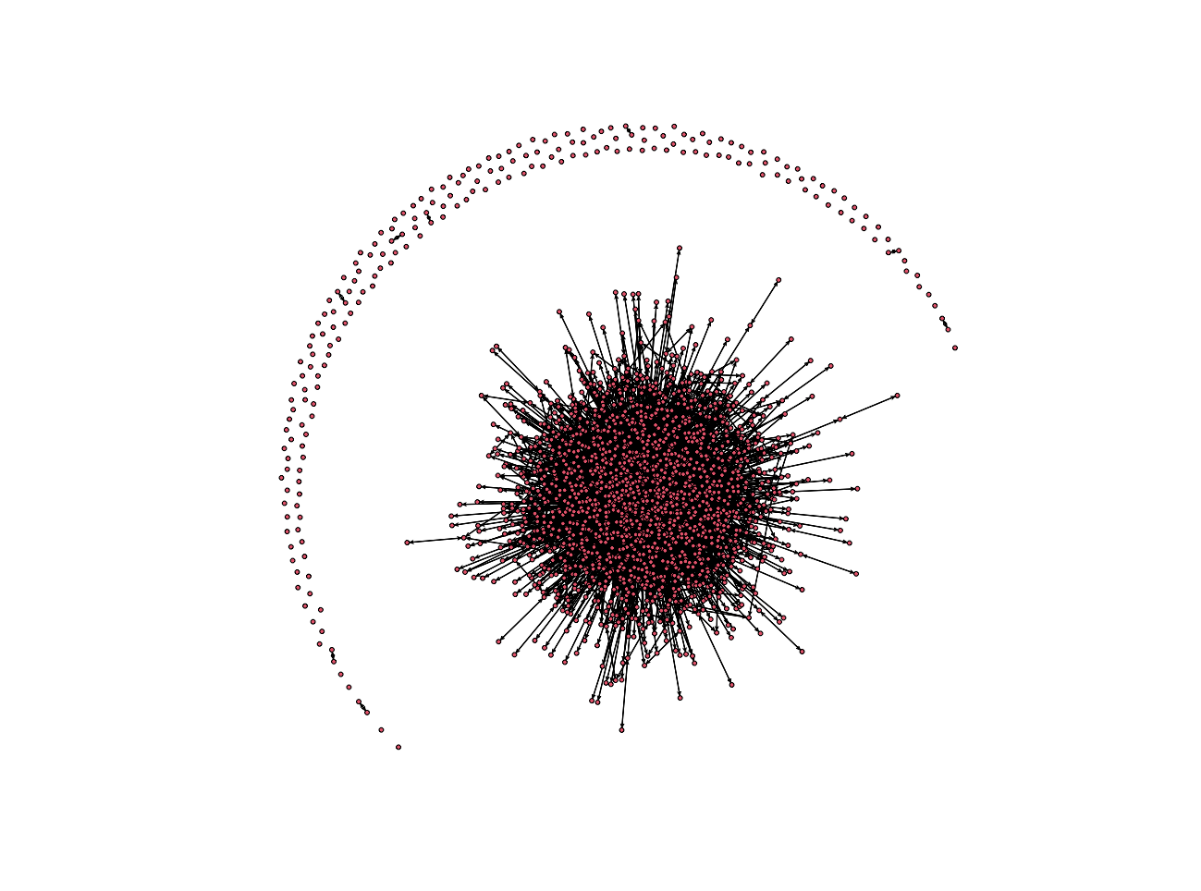
**

**Figure S2.** Example of higher degree network.

A) Network structure B) distribution of main partners C) number of partners by quantile

**
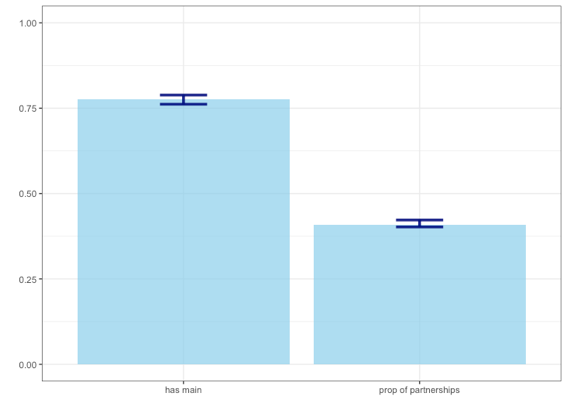
A B**

**C**

**
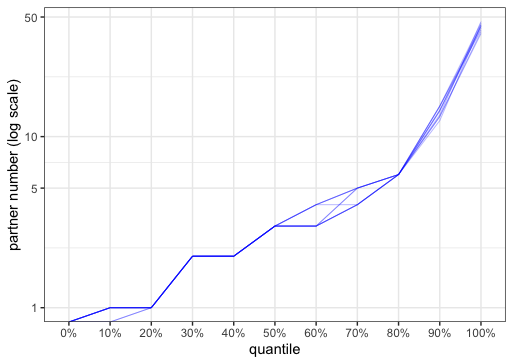
**

**Common random numbers (CRN)**

CRN was applied to all individual- and tie-level events. There were seven *i) individual-level events* and two *ii) tie-level events*. In addition, there was a probability of *iii) infection acquisition from one-off partnerships*. We created a random number matrix of random numbers between 0-1 referred to as master CRN matrix from hereon. The master CRN matrix has 10^5 rows and 417 columns (number of timesteps in the model to simulate 8 years with weekly timestep). Event-specific CRN matrices were drawn from the master CRN matrix; operationalized by randomly sampling of 2,000 row numbers (representing the population size) with replacement for each event and using the row numbers to index the master CRN matrix in the model simulations. The master CRN matrix, and accessing it via row indices, improves efficiency and reduces computational needs during calibration of the model and subsequent scenario analyses.

This approach ensures variation at the event, individual, and timestep level when the model is repeatedly simulated with the same parameter set but different draws from the master CRN matrix, but it provides identical results when the same combination of parameters and CRN are used. This allows for improved evaluation of counterfactual scenarios, such as increased levels of partner notification modeled in this study. In the counterfactual scenarios for a given parameter set and CRN combination, the changes observed in outcomes (between the baseline and counterfactual simulation) are caused by the change in partner notification.

***i) Individual-level events***

CRN matrices were applied to individual-level events as sampling with replacement 2,000 rows from the master CRN matrix for each event. There were seven individual-level events modeled this way, each represented by unique 2,000x417 CRN matrix drawn for each simulation:

- probability of developing symptoms after infection
- probability of screening if asymptomatic infection
- probability of testing if symptomatic infection
- probability of natural clearance
- probability of partner notification if index case is identified
- which partner is notified, if index case notifies a partner
- probability of engaging in one-off partnership in a given timestep

For example, for the parameter governing weekly clearance probability, among individuals who were infected in the previous timestep (*i-1)*, we evaluate the CRN matrix for clearance against clearance probability for individual *m*, at timestep *i*, and if

*CRN_clearance_[m,i] < probability of clearance*,

the individual recovers from infection at timestep *i,* and moves to susceptible health state. Similar approach is applied to all individual-level events.

For assigning which partner is notified, we use an event-specific CRN matrix, and information on the number of main partners an individual *m* has: if there are x number of main partners, they are assigned evenly spaced adjacent ranges from 0-1 and the partner whose range contains the value in *CRN_notified_[m,i]* is selected for partner notification. This in turn ensures that the same partner is notified were the same simulation run repeatedly with the same parameter set.

***ii) Tie-level events***

For tie-level events (weekly transmission probability from main or casual partner), we draw a CRN matrix separately for casual and main ties in the same fashion*: CRN_casual_ties_[m,p,i]*, where *m* is the individual, *p* are *m*’s casual ties, and *i* is the time step. At each timestep, for ties where *m* is susceptible and person *p* is infected, transmission probability is evaluated and occurs for person *m* if

*CRN_casual_ties_[m,p,i]* *< transmission probability for casual partnerships*

***iii) Infection acquisition from one-off partnerships***

For one-off partnerships, if person *m* is susceptible and engages in one-off partnership in timestep *i*, the probability of infection is evaluated as:

*Probability of infection[m,i]=1-(1-transmission probability per act* prevalence of infection in those with one-off partners)^number of acts*.

If *CRN_one_off[_[m,i] < probability of infection[m.i],* person *m* changes health states from susceptible to infected at time *i.*

**Simulation number needed**

To obtain stable effect sizes when results are averaged at the parameter set level, we tested the number of simulations needed using 20% relative increase in partner notification (PN) as a counterfactual scenario. We compared this against an identical model without CRN to estimate the benefits gained from implementing CRN. We determined we needed 80 simulations per parameter draw to obtain stable impact estimates by comparing the results from simulations with increasing number of simulations (Figure S3). Comparison against model simulations without CRN demonstrated the reduced variance in simulations where CRN is employed.

**Figure S3.** Estimated effect size when the model simulations are pooled together using increasing simulation sample size (x-axis). Blue summary estimates present simulations where CRN was not employed and red summary estimates are simulations where CRN was used. The vertical line shows the effect size when 280 simulations are used (with CRN).


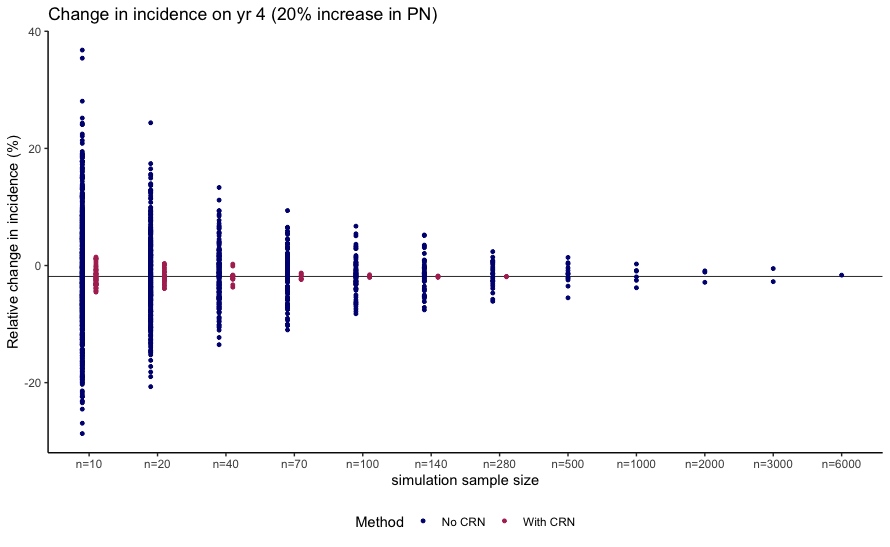


**Implementation of partner notification**

Partner notification requires:

1. an infected index case to be tested and diagnosed (testing due to symptoms, or via screening of asymptomatic individuals)
2. one or more of the partners of the index case to be notified (via patient- or provider-initiated partner notification)
3. for the partner to be tested and, if they are infected, treated (or in the case of expedited partner therapy [EPT], provided presumptive treatment)

We define partner notification (PN) coverage at the population level as a function of i) and ii), and our analysis assumes that if the notified partners are infected, they receive curative treatment. PN coverage is defined as a cumulative estimate:

$PN coverage=\frac{cumulative number of partners notified}{cumulativve number of index cases diagnosed}$ .

The numerator reflects the number of partners notified in the population *irrespective of whether those individuals were infected* at the time they received PN. The denominator includes diagnosed cases identified via symptomatic testing or asymptomatic screening and does not include the number of people diagnosed due to partner notification; we do not model partners identified via PN notifying their partners.

PN coverage is operationalized in the model as a dynamic process dependent on the number of index cases diagnosed: for individual *m* who is diagnosed at time *i*, partner notification occurs if population level cumulative PN coverage < target PN coverage and, if person *m* has main partners, then one of person *m*’s main partners receives PN. In case there are >1 main partners, the partner notified is drawn at random. For this, we use CRNs (see section on common random numbers: individual-level events). At baseline, as part of model calibration, we vary PN coverage within plausible bounds, and in the counterfactual scenarios, we increase the PN coverage from baseline levels.

**Regression model for univariate sensitivity analysis**

We used linear regression with generalized estimating equations to estimate the contribution of different drivers to incidence ratio ratio (IRR). Parameter set was used as the cluster ID. We employed the R package glmtoolbox^3^ At the simulation-level, the following model was used:

glmgee(RR_incidence_~. , id=id, family = gaussian, corstr = "exchangeable", data=df_sall)

The same analysis was repeated at the parameter set level, where an average of simulation-level results was taken if the variables varied at the simulation level:

lm(RR_incidence_ ~. , data = df_all)

**Model calibration**

Target fitting (Table S1) ranges used (per person)

1. Prevalence: between 0.02-0.1
2. Incidence: between 0.01 – 0.23
3. Diagnosis rate: between 0.01-0.1
4. Proportion of symptomatic: between 0.4-0.8

We calibrate the model by running each parameter set 10 times, and calculating the average of calibration outcomes after 4 years of burn in.

**Table S1.** Calibration targets used

| **Variable** | **Ranges** | **Data and references** |
| --- | --- | --- |
| **Gonorrhea prevalence** | 2-10 % | In 2011-2012, SSuN urogenital gonorrhea positivity in MSM was 11.1%, (7.9%) tested positive for pharyngeal gonorrhea, and 1136 (10.2%) tested positive for rectal gonorrhea. ^4^ SSuN data represents people who were tested at STD clinics, and likely overestimates prevalence. The lower limit is defined based on gonorrhea prevalence in nationally representative sample of sexually active MSM.^5^ Modeling study in Baltimore and San Francisco estimated gonorrhea prevalence among MSM to be between 4-10%^6^ |
| **Gonorrhea diagnosis rate**  Defined in the model as annual diagnosis rate over total population | 1-10% | National HIV Behavioral Surveillance (27 cities in 2017) had 10.8% of HIV-negative MSM self-report gonorrhea diagnosis in the past 12 months.^7^ We considered this to represent the highest plausible estimate given the sampling of MSM was based on recruitment in venues. |
| **Proportion of gonorrhea which are symptomatic at diagnosis**  Defined in the model as proportion of diagnoses which occurred in people with symptoms | 30-80% | Proportion of diagnoses which are symptomatic varied in Baltimore and San Francisco, and were observed to vary by age, and we defined broad ranges for this target.^6^ |
| **Gonorrhea incidence**  Defined as annual incidence rate over total population | 2-23% | We defined a plausible interval for incidence as between 2 and 23% based on two cohorts in Atlanta and San Francisco with upper level allowed to be higher than estimated assuming that some incident gonorrhea may be missed when testing interval is several months. In San Francisco, MSM were tested every 6 months. Yearly incidence of rectal gonorrhea was estimated at 3.5% (1.6-7.0%), urethral gonorrhea 1.5% (0.6-3.4%), and pharyngeal gonorrhea 11.7% (8.8-15.3%).^8^ Studies by Kelley et al (2015)^9^ and Sullivan et al (2014)^10^ present data from a prospective cohort 2011-2014, restricted to MSM without HIV. Incidence per 100 person years for rectal gonorrhea was 9.4 (6.3-13.4) among Black men, and 3.7 (2.1-6.1) among White men. Urethral gonorrhea incidence was 2.2 (0.9-4.3) among Black men and 0.2 (0.0-1.2) among White men. |

.

**RESULTS**

**Calibration results**

The figures show the calibration ranges in red, simulations in grey (1000 randomly selected simulations shown), and the average of simulations by parameter set in blue. Included are the parameter sets which were included in the final analysis. Prevalence is shown as the weekly prevalence per person, and incidence, diagnoses and proportion symptomatic are shown as annual number of events per person.

**Figure S4.** Included simulations for prevalence (A), incidence (B), diagnosis (C), and proportion symptomatic (D)


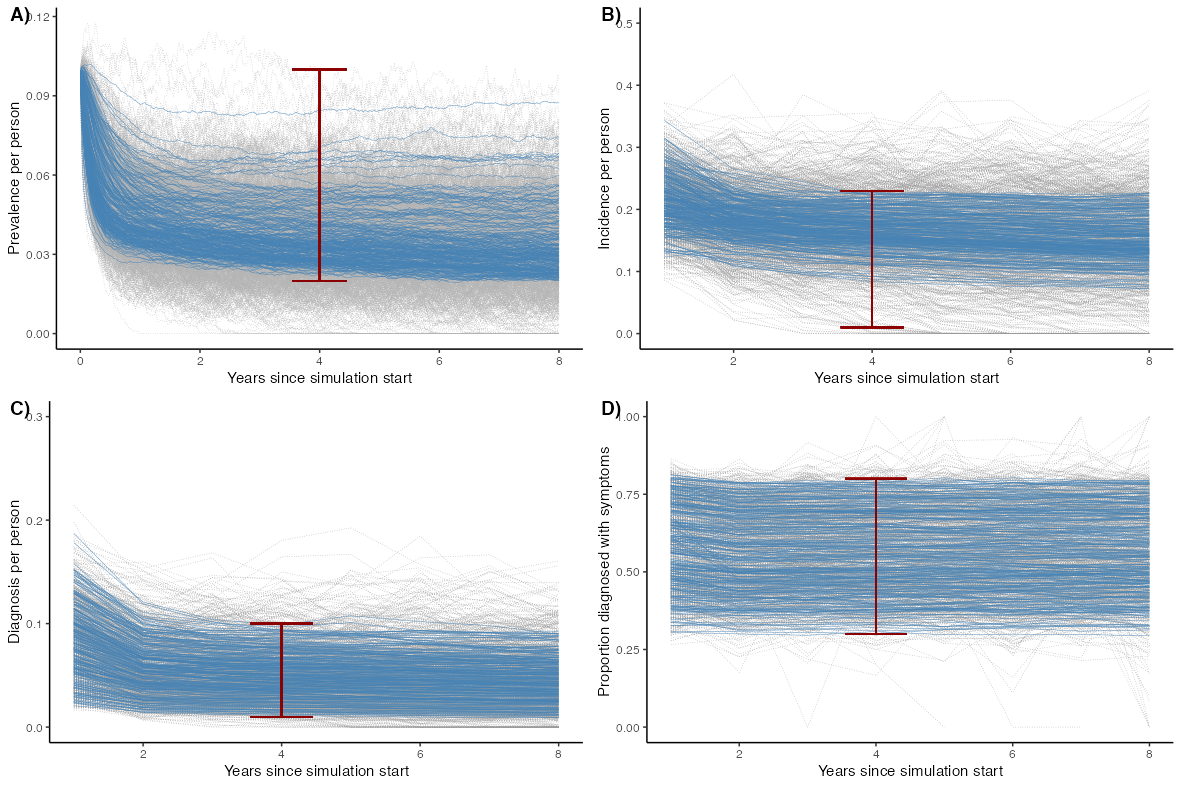


**Figure S5.** Model parameters where dark orange dashed-line distribution describes the prior distribution, and blue line the distribution in the calibrated parameter sets. These correspond to the 12 parameters in Table 1 in the main manuscript, and the PN coverage which is derived from the two PN parameters.

**
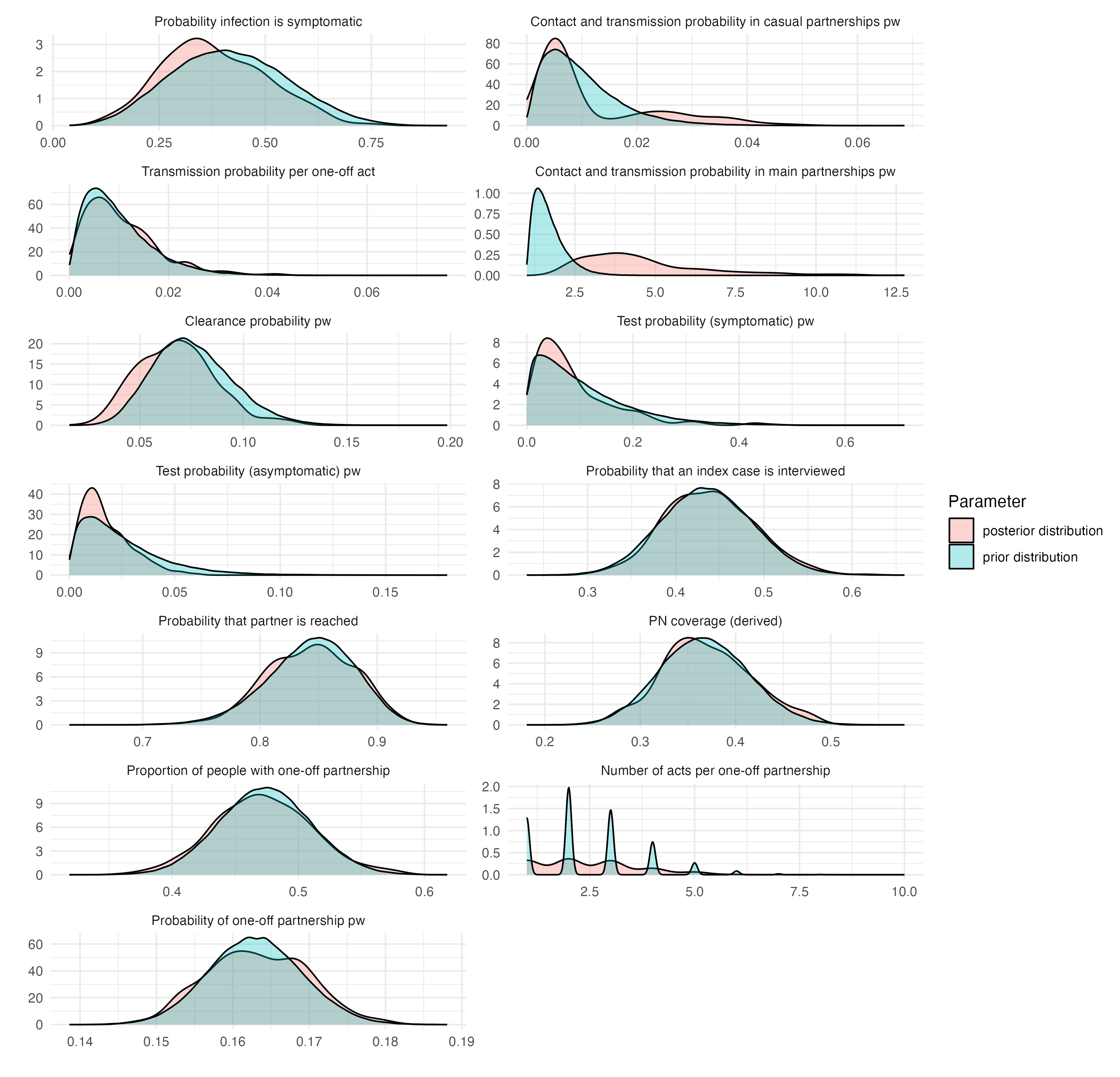
**

pw: per week

**Additional figures from the results**

**Figure S6.** Correlation of variables included in the regression model

**
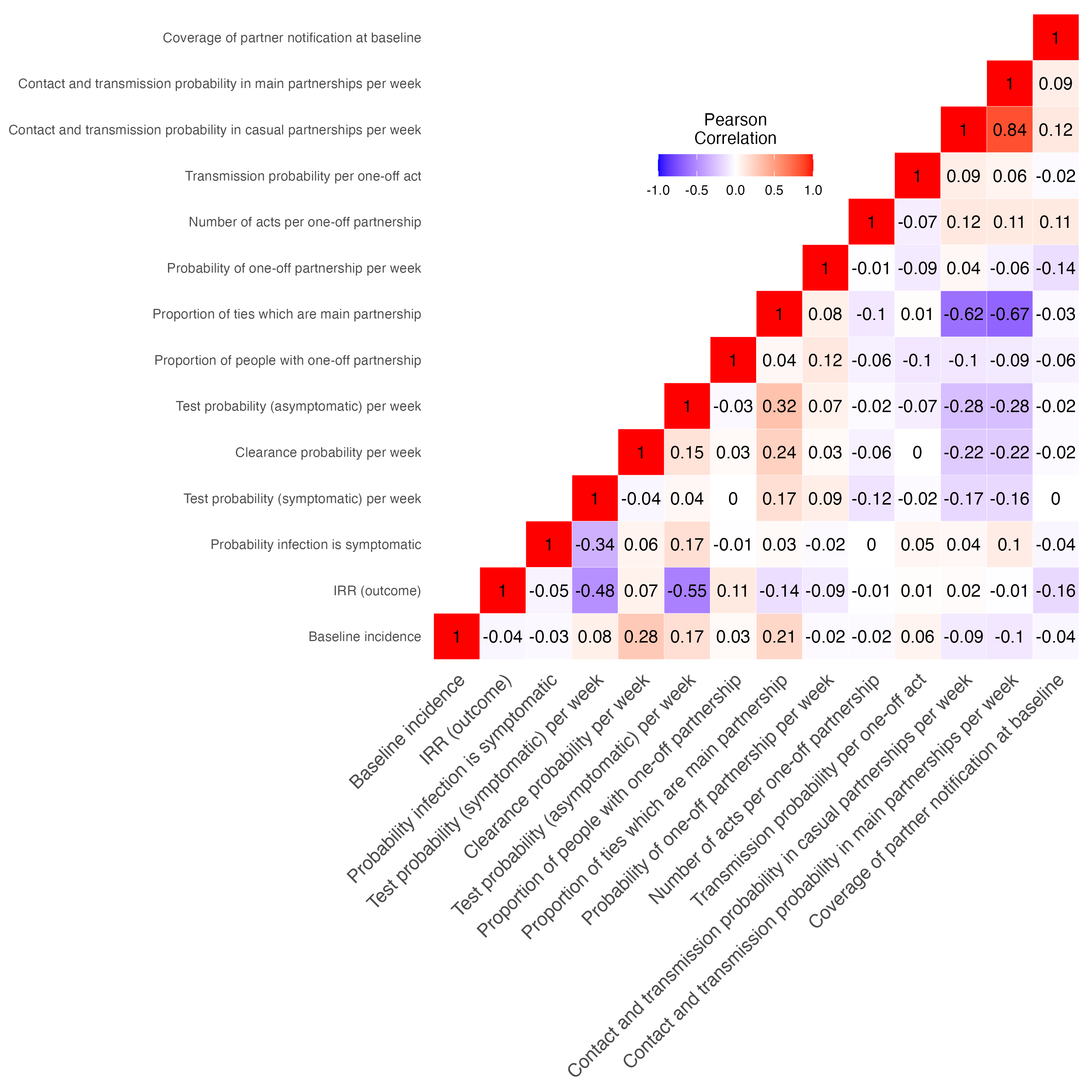
**

**Figure S7.** Number of people in networks by their partner (main and casual) number.


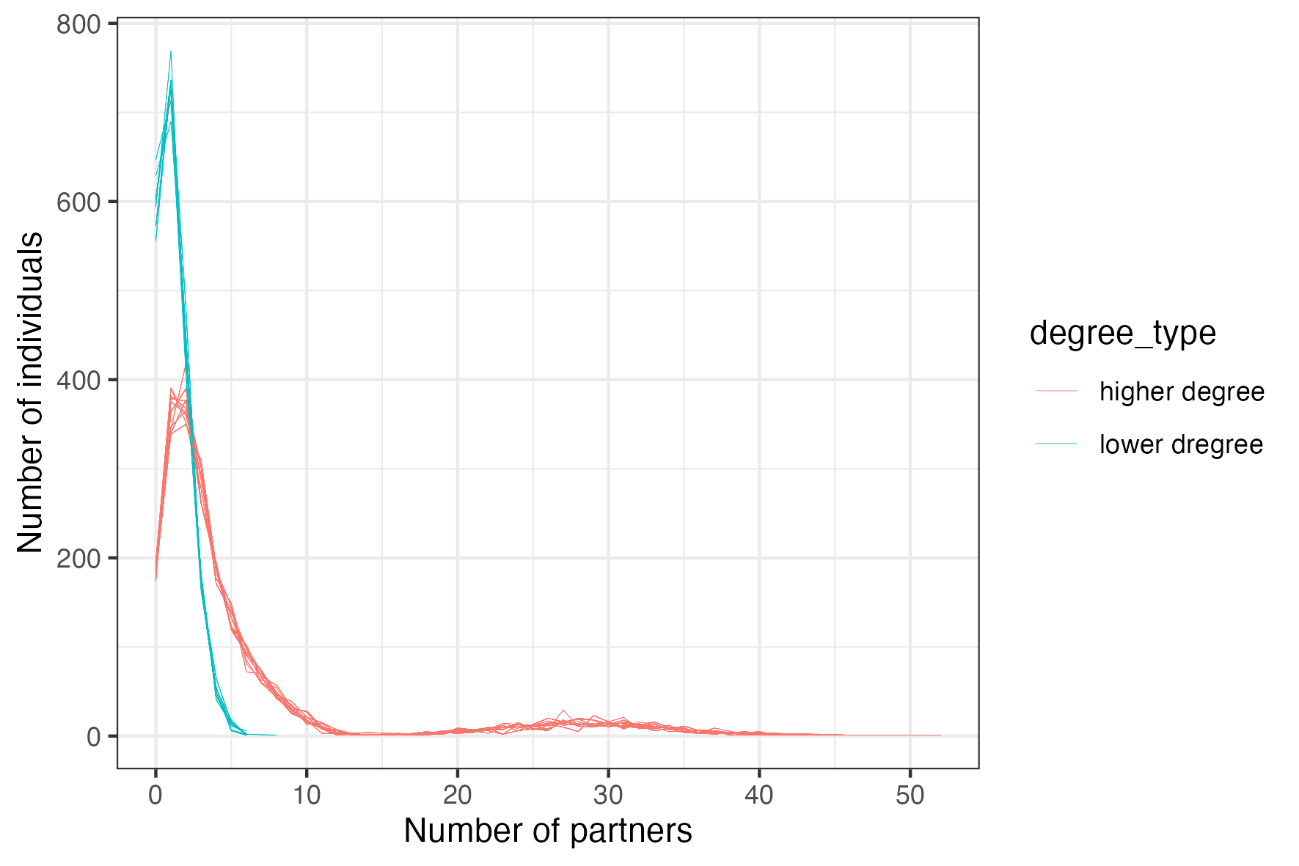


**References**

1. Weiss KM, Goodreau SM, Morris M, Prasad P, Ramaraju R, Sanchez T, et al. Egocentric sexual networks of men who have sex with men in the United States: Results from the ARTnet study. Epidemics. 2020 Mar 1;30:100386.

2. National Opinion Research Center U of C. General Social Survey [Internet]. [cited 2023 Oct 17]. Available from: https://gss.norc.org/

3. Set of Tools to Data Analysis using Generalized Linear Models [R package glmtoolbox version 0.1.9]. 2023 Oct 10;

4. Patton ME, Kidd S, Llata E, Stenger M, Braxton J, Asbel L, et al. Extragenital gonorrhea and chlamydia testing and infection among men who have sex with men-STD Surveillance Network, United States, 2010-2012. Clinical Infectious Diseases. 2014;58(11):1564–70.

5. Centers for Disease Control and Prevention (CDC). National Center for Health Statistics (NCHS). National Health and Nutrition and Examination Survey Data. [Internet]. Hyattsville, MD: U.S. Department of Health and Human Services, Centers for Disease Control and Prevention; [cited 2019 May 5]. Available from: https://wwwn.cdc.gov/nchs/nhanes/default.aspx

6. Rönn MM, Testa C, Tuite AR, Chesson HW, Gift TL, Schumacher C, et al. The potential population-level impact of different gonorrhea screening strategies in Baltimore and San Francisco. Sex Transm Dis. 2019 Dec;1.

7. Wortley P, Todd J, Melton D, Masiello Schuette S, Kern D, Jimenez Dallas AD, et al. HIV Infection Risk, Prevention, and Testing Behaviors Among Men Who Have Sex With Men—National HIV Behavioral Surveillance, 23 U.S. Cities, 2017. 2019.

8. Morris SR, Klausner JD, Buchbinder SP, Wheeler SL, Koblin B, Coates T, et al. Prevalence and Incidence of Pharyngeal Gonorrhea in a Longitudinal Sample of Men Who Have Sex with Men: The EXPLORE Study. Clinical Infectious Diseases. 2006 Nov 15;43(10):1284–9.

9. Kelley CF, Vaughan AS, Luisi N, Sanchez TH, Salazar LF, Frew PM, et al. The Effect of High Rates of Bacterial Sexually Transmitted Infections on HIV Incidence in a Cohort of Black and White Men Who Have Sex with Men in Atlanta, Georgia. AIDS Res Hum Retroviruses. 2015 Jun;31(6):587–92.

10. Sullivan PS, Peterson J, Rosenberg ES, Kelley CF, Cooper H, Vaughan A, et al. Understanding racial HIV/STI disparities in black and white men who have sex with men: a multilevel approach. PLoS One. 2014;9(3):e90514.
